# Encephalopathy associated with autoimmune thyroid disease in patients with Graves' disease: clinical manifestations, follow-up, and outcomes

**DOI:** 10.1186/1471-2377-10-27

**Published:** 2010-04-28

**Authors:** Gianluca Tamagno, Yahya Celik, Rafael Simó, Marcel Dihné, Kazumi Kimura, Giorgio Gelosa, Byung I Lee, Caroline Hommet, Giovanni Murialdo

**Affiliations:** 1 Department of Endocrinology and Diabetes Mellitus, St Vincent's University Hospital, University College Dublin, Dublin, Ireland; 2 Department of Neurology, Trakya University School of Medicine, Edirne, Turkey; 3 Division of Endocrinology, Vall d'Hebron University Hospital, Barcelona, Spain; 4 Department of Neurology, University Hospital Düsseldorf, Düsseldorf, Germany; 5 Department of Stroke Medicine, Kawasaki Medical School, Okayama, Japan; 6 Department of Neurology, San Gerardo Hospital, University of Milano-Bicocca, Monza, Italy; 7 Department of Neurology, Severance Hospital, Yonsei University College of Medicine, Seoul, Korea; 8 Service of Geriatric Internal Medicine, Regional University Hospital, Tours, France; 9 Department of Endocrine and Medical Sciences, University of Genoa, Genoa, Italy

## Abstract

**Background:**

The encephalopathy associated with autoimmune thyroid disease (EAATD) is characterized by neurological/psychiatric symptoms, high levels of anti-thyroid antibodies, increased cerebrospinal fluid protein concentration, non-specific electroencephalogram abnormalities, and responsiveness to the corticosteroid treatment in patients with an autoimmune thyroid disease. Almost all EAATD patients are affected by Hashimoto's thyroiditis (HT), although fourteen EAATD patients with Graves' disease (GD) have been also reported.

**Methods:**

We have recorded and analyzed the clinical, biological, radiological, and electrophysiological findings and the data on the therapeutic management of all GD patients with EAATD reported so far as well as the clinical outcomes in those followed-up in the long term.

**Results:**

Twelve of the fourteen patients with EAATD and GD were women. The majority of GD patients with EAATD presented with mild hyperthyroidism at EAATD onset or shortly before it. Active anti-thyroid autoimmunity was detected in all cases. Most of the patients dramatically responded to corticosteroids. The long term clinical outcome was benign but EAATD can relapse, especially at the time of corticosteroid dose tapering or withdrawal. GD and HT patients with EAATD present with a similar clinical, biological, radiological, and electrophysiological picture and require an unaffected EAATD management.

**Conclusions:**

GD and HT equally represent the possible background condition for the development of EAATD, which should be considered in the differential diagnosis of all patients with encephalopathy of unknown origin and an autoimmune thyroid disease, regardless of the nature of the underlying autoimmune thyroid disease.

## Background

Encephalopathy associated to autoimmune thyroid disease (EAATD), also called Hashimoto's encephalopathy, is a rare condition that may occur in patients with clinical or sub-clinical autoimmune thyroid disease. It is characterized by unspecific and protean neurological and/or psychiatric symptoms often associated with high serum and/or cerebrospinal fluid (CSF) levels of anti-thyroid antibodies (Abs), increased CSF protein concentration, non-specific diffuse electroencephalogram (EEG) abnormalities, and responsiveness to the treatment with corticosteroids [1-4]. The diagnosis of EAATD is still based mostly on exclusion criteria, which might affect the accurate estimation of its genuine prevalence.

Several mechanisms, like cerebral autoimmune vasculitis with focal or global brain hypoperfusion, cerebral tissue-specific autoimmunity with or without demyelination, and neuronal dysfunction secondary to brain edema have been thought to be involved in the pathogenesis [1,2,5-12]. Generally, EAATD occurs in patients with normal, or slightly abnormal, thyroid hormone levels and seems to be unrelated to the thyroid function [2,4,8]. Anti-thyroperoxidase (TPO) and anti-thyroglobulin (TG) Abs have been often detected in the CSF of EAATD patients but their possible role in the pathogenesis has been not defined [2]. Novel antigens, like α-enolase and a 36-kDa protein present in a soluble fraction from the cerebral cortex, have been recently identified in EAATD patients but their involvement in the pathogenesis has been not documented enough [9,10]. Converging evidences support the hypothesis of EAATD as a cerebral autoimmune vasculitis with or without immune-complex deposition [3,6].

The clinical manifestations range from stroke-like focal signs to generalized symptoms, either with dramatic or blunted presentation. Seizures, loss of consciousness, cognitive alterations, hallucinations and psychiatric disorders, behavioral changes, myoclonus, involuntary movements including tremors, ataxia, language impairment, sensory deficits, headache, and inflammatory signs of encephalitis and/or meningitis have been frequently reported [2,4,11,12].

The onset of EAATD may be acute or sub-acute and the following trend progressive or relapsing. By definition, EAATD symptoms are steroid-responsive, but spontaneous remission or lack of responsiveness to corticosteroids rarely occurs. The prognosis appears depending on the responsiveness to the corticosteroid treatment but the evolution of the disease in the long-term is unpredictable.

Almost all patients with EAATD present with Hashimoto's thyroiditis (HT) as the background autoimmune thyroid disease. Only a small number of EAATD patients with a diagnosis of Graves' disease (GD) have been reported to date [3,13-24]. We hereby review the full case series of EAATD patients with GD published so far and report the clinical manifestations, the evolution in the long-term, and the outcomes in these patients.

## Methods

The cases of EAATD in patients with GD published in the international medical literature up to 2009, August 31^st ^have been searched by the Internet scientific research engine PubMed. "Hashimoto's encephalopathy", "encephalopathy associated to/with autoimmune thyroid disease", "encephalopathy", "Graves' disease", and "hyperthyroidism" have been used as both isolated and crossed keywords. No limits have been imposed to the research. A total number of 83788 published papers (all article types) were found. Following review of the abstracts or the full texts, the majority of the papers were excluded (papers appearing repetitively at the PubMed searches, falling out of topic, reporting cases of EAATD in HT, reporting cases of GD without EAATD, or reporting research in animals or in vitro solely). Finally, 13 papers that describe and characterize cases of EAATD occurred in patients with a defined diagnosis of GD were identified, accounting for a total of fourteen patients [3,13-24]. In the cases detected, the diagnosis of GD and EAATD were done according to the current criteria. Features compatible with EAATD without a defined diagnosis of the disease were disclosed in three GD patients [25,26]. Language limitations (Japanese, Slovak) and availability of the abstract only did not allow the proper data collection for confirming GD diagnosis and reporting exhaustively the clinical and biological features of two patients [27,28]. These five patients have been not listed in our case series.

The data on the main anthropometric features, thyroid function, serum and CSF levels of anti-TPO, anti-TG, and anti-TSH Abs, imaging and EEG findings, and EAATD symptoms have been collected and analyzed. The personal collaboration and the confidential data exchange among the authors allowed the data collection and analysis.

In the patient series, the concomitant presence of other pathological conditions potentially linked to thyroid or systemic autoimmunity has been checked. When possible, the patients were re-evaluated in the follow-up. In such cases, the length of the follow-up and the clinical and therapeutic outcomes were recorded, including data about EAATD reoccurrences. The numerical data was calculated as mean ± standard deviation and range (patient age, duration of the follow-up, duration of the corticosteroid treatment) or rates (EAATD symptoms, biological findings). A comparison of the most relevant immunological, CSF, magnetic resonance imaging (MRI), and EEG findings reported in GD patient series versus a homogeneous series of HT patients [12] with EAATD was performed and two-tailed Fisher exact test has been used for the statistical analysis.

## Results

### Anthropometric and clinical features

Fourteen patients with a defined diagnosis of GD and EAATD have been recorded (Table 1), of which twelve were females. Patient mean age at EAATD onset was 43.8 ± 19.7 years (range: 12-79 years). Altered consciousness (12/14), involuntary movements (including tremor and myoclonus) (8/14), seizures (7/14), and cognitive impairment (6/14) were the most frequently reported symptoms. Sensorial alterations, headache, focal symptoms, ataxia, language impairment, signs of encephalitis, and psychiatric symptoms have been also described. All patients but one, which spontaneously improved, were treated with corticosteroids and they showed a dramatic improvement of EAATD symptoms with the exception of two cases (Patient 7 responded partially, Patient 8 did not respond and required administration of intravenous immunoglobulins). Patient 4 had plasmapheresis associated to the administration of corticosteroids. Methylprednisolone 1000 mg daily (or equivalent) as attack dose for a few days followed by oral corticosteroids with a slow and progressive tapering of the daily dose was the most used treatment outline.

**Table 1:** Patients with Graves' disease and encephalopathy associated with autoimmune thyroid disease.

Reference	Age	Gender	Clinical manifestations	EAATD relapses (in the short term)	Thyroid hormones	Responsiveness to corticosteroid therapy
**Patient 1 **[3]	21	♀	seizures, involuntary movements, altered consciousness	no	- hyperthyroidism	- clinical improvement - decrease of the auto-Abs

**Patient 2 **[13]	23	♀	seizures, cognitive impairment, altered consciousness	1 relapse	- hyperthyroidism - euthyroidism at EAATD relapse	- clinical improvement - decrease of the auto-Abs

**Patient 3 **[14]	51	♀	involuntary movements, cognitive impairment, decreased verbal fluency, altered consciousness	1 relapse	- hyperthyroidism - euthyroidism at EAATD relapse	- clinical improvement - decrease of the auto-Abs

**Patient 4 **[15]	45	♀	involuntary movements, mono-lateral weakness, dysarthria, altered consciousness, hallucinations, sphincters incontinence	no	- hyperthyroidism	- clinical improvement - decrease of the auto-Abs - plasmapheresis was required

**Patient 5 **[16]	29	♀	headache, altered hearing, nausea and vomit, blunted papilla, nystagmus	no	- hyperthyroidism	- clinical improvement - decrease of the auto-Abs

**Patient 6 **[17]	57	♀	involuntary movements, altered consciousness	no	not reported	- clinical improvement

**Patient 7 **[17]	25	♀	seizures, headache, involuntary movements, ataxia, aphasia, altered consciousness	persisting symptoms	not reported	- partial improvement

**Patient 8 **[18]	12	♀	optic neuritis, dysmetria, dysdiadochokinesis	no	- hyperthyroidism	- clinical worsening

**Patient 9 **[19]	68	♀	seizures, altered consciousness, anxiety, hallucinations	no	- euthyroidism	- clinical improvement

**Patient 10 **[20]	61	♂	involuntary movements, ataxia, cognitive impairment, altered consciousness, depression	no	- post-RAI hypothyroidism	- clinical improvement

**Patient 11 **[21]	47	♀	seizures, headache, hemiplegia, cognitive impairment, altered consciousness, hallucinations	no	- hyperthyroidism	- clinical improvement - decrease of the auto-Abs

**Patient 12 **[22]	40	♀	seizures, hemiplegia, dysathria, cognitive impairment, altered consciousness	no	- hyperthyroidism	- no corticosteroid treatment - spontaneous improvement

**Patient 13 **[23]	79	♀	seizures, involuntary movements, cognitive impairment, altered consciousness	not reported	- hyperthyroidism	- clinical improvement

**Patient 14 **[24]	55	♂	involuntary movements, ataxia, altered consciousness, anxiety, hallucinations	1 relapse	- hyperthyroidism - very mild hypothyroidism at EAATD relapse	- clinical improvement - decrease of the auto-Abs

The main anthropometrics data, biological findings, and clinical features of the fourteen patients with a defined diagnosis of Graves' disease and encephalopathy associated with autoimmune thyroid disease (EAATD) reported in the literature so far (Abs: antibodies; RAI: radioactive iodine).

Only 4/14 cases of Graves' ophthalmopathy have been reported (Patient 3, 4, 10, and 12). No other GD-related clinical manifestations, including myopathy and dermopathy, were described. None of the patients presented with other autoimmune diseases, like gastritis and arthritis prior to EAATD occurrence. After the diagnosis of EAATD, Patient 10 developed acquired autoimmune hemophilia with anti-factor VIII Abs despite the concomitant corticosteroid treatment and thereafter was treated also with cyclophosphamide.

### Biological findings

Evidence of mild/moderate or subclinical hyperthyroidism at EAATD onset, or a few weeks before it, was reported in all patients except Patient 12, euthyroid, and Patient 10, with post-radioactive iodine hypothyroidism not adequately replaced. All patients had evidence of active anti-thyroid autoimmunity in their serum samples but the complete panel of anti-thyroid Abs (anti-TG, anti-TPO, and anti-TSH receptor Abs) was assessed in seven patients only (Patient 1, 3, 5, 10, 11, 12, and 13). Serum anti-TPO Abs were positive in all cases (14/14). Serum anti-TG and anti-TSH receptor Abs were positive in 7/11 (Patient 1, 3, 4, 5, 6, 7, and 13) and 9/10 (Patient 1, 2, 3, 5, 8, 11, 12, 13, and 14) patients, respectively. In the CSF, anti-TPO and anti-TG Abs were positive in 5/5 (Patient 2, 6, 7, 10, and 14) and 2/2 (Patient 6 and 7) cases, respectively. Anti-TSH receptor Abs were assayed in the CSF of Patient 5 only with negative result. The CSF protein concentration was increased in 7/11 patients (Patient, 1, 2, 3, 4, 10, 11, and 14). In Patient 9, the CSF protein level ranged inside the reference limits but the IgG and albumin CSF/serum ratios were slightly elevated. In five patients, mild lymphocytic pleocytosis was disclosed (Patient 4, 5, 8, 9, and 10).

### Imaging and EEG findings

Brain computed tomography (CT) was normal in 3/4 (Patient 5, 12, and 13) but showed an inflammatory pattern in Patient 9. MRI showed normal or not relevant findings in 9/13 patients (Patient 1, 2, 3, 4, 6, 9, 11, 13, and 14). Patient 5 presented with non-specific signal changes of the white matter, cerebral edema, and parenchyma alterations compatible with inflammation. Isolated hyperintense white matter changes were seen in 3/12 patients and were reversible (Patient 8, 10, and 12). In Patient 4, the brain MRI was normal but a severe stenosis of the internal carotid arteries and arteries distal to the basilar artery was revealed by magnetic resonance angiography and improved after EAATD treatment. However, the angiogram was normal in Patient 12 who had abnormal MRI findings. A global reduction of brain perfusion was demonstrated by single photon emission computed tomography (SPECT) in Patient 2 and Patient 13, both having unremarkable MRI findings. In Patient 3, positron emission tomography revealed a diffuse hypometabolic cerebral pattern that recovered after the EAATD symptoms disappeared. The EEG records were abnormal in 12/13 patients (Patient 1, 2, 3, 4, 5, 7, 9, 10, 11, 12, 13, and 14). A diffuse and not specific slowing of the background EEG activity was the most frequent record (Patient 1, 2, 3, 4, 5, 10, 11, 12, and 14).

### Follow-up

Patient 2, Patient 3, and Patient 14 showed a relapse of EAATD in the short-term clinical course. These events occurred with a concomitant euthyroid state under anti-thyroid therapy in the first two patients and iatrogenic very mild hypothyroidsm in Patient 14. In Patient 2, EAATD relapsed one month after the first episode. In the other two cases, the recurrence of EAATD symptoms was concomitant with the early corticosteroid dose tapering (Patient 3) or withdrawal (Patient 14) and was promptly reversed by restoring an effective daily corticosteroid dose in the first case and thyroidectomy in the second one. Both patients experienced the relapse about two weeks after the first EAATD occurrence.

Original data regarding the long-term follow-up and outcomes have been recorded in six patients (Patient 1, 4, 5, 10, 12, and 14) (Table 2). Two patients (Patient 3 and Patient 9) were lost at the long-term follow-up. There is no data about the lasting six patients available (Patient 2, 6, 7, 8, 11, and 13). The average follow-up duration of the six patients fully described in the long-term was 37.5 ± 11.0 months (range: 26-54 months). After the recovery from the EAATD episode, only patient 10 had a relapse (month 21). This event was concomitant to the corticosteroid dose tapering. The corticosteroid therapy was beneficial in 4/4 patients receiving such a treatment in the long-term and was withdrawn in three of them in 14.0 ± 8.5 months (range: 6-23 months). Patient 10 did not stop his corticosteroid treatment. On the contrary, Patient 12 did not receive corticosteroids because of spontaneous improvement of EAATD and experienced no relapses up to her last follow-up visit (month 26). Patient 14 did not receive any corticosteroid treatment after total thyroidectomy and remained asymptomatic during the follow-up so far (month 30). GD relapsed in 2/6 patients (Patient 1 and Patient 5) and radioiodine and total thyroidectomy were needed, respectively. During the follow-up, neither classical extra-thyroid GD manifestations nor other autoimmune diseases were reported in any patients but Patient 10 who developed autoimmune hemophilia (month 24). Actually, Patient 10 was the only case of EAATD relapse in the long-term follow-up, occurring three months before the report of autoimmune hemophilia. The combination of the above conditions required a combined treatment with corticosteroids and cyclophosphamide, both still ongoing at his last follow-up visit (month 33).

**Table 2:** Long-term follow-up of eight patients with Graves' disease and encephalopathy associated with autoimmune thyroid disease.

Reference	Follow-up (months)	Anti-thyroid Abs (serum)	GD relapses	EAATD relapses	Other autoimmune manifestations	Treatment
**Patient 1 **[3]	34	- normal anti-TG, anti-TPO, and anti-TSH Receptor Abs	1 relapse (month 18)	no	no	- prednisone (from month 1 to month 23) - RAI (month 20) - levothyroxine (from month 24)

**Patient 3 **[14]	lost at follow-up					

**Patient 4 **[15]	48	- normal anti-TG Abs - increased anti-TPO Abs	no	no	no	- prednisone (from month 1 to month 13) - propylthiouracil (from month 1 to month 4 and from month 18 to 40) - radioiodine therapy (month 6) - total thyroidectomy (month 32)

**Patient 5 **[16]	54	- normal anti-TG, anti-TPO, and anti-TSH Receptor Abs	1 relapse (month 20)	no	no	- prednisone (from month 1 to month 6) - propylthiouracil (from month 1 to month 18 and from month 20 to 45) - total thyroidectomy (month 45) - levothyroxine (from month 45)

**Patient 9 **[19]	lost at follow-up					

**Patient 10 **[20]	33	- normal anti-TG and anti-TSH Receptor Abs - increased anti-TPO Abs	no	1 relapse (month 21)	autoimmune hemophilia (anti-factor VIII Abs +)	- levothyroxine (starting before EAATD occurrence) - prednisolone (from month 1) - cyclophosphamide (from month 24)

**Patient 12 **[22]	26	- normal anti-TG Abs - increased anti-TPO and anti-TSH Receptor Abs	no	no	no	- methimazole (from month 1)

**Patient 14 **[24]	30	- normal anti-TG Abs - increased anti-TPO and anti-TSH Receptor Abs	no	no	no	- methylprednisolone (month 1) - total thyroidectomy (month 1) - levothyroxine (from month 1)

Anti-thyroid antibodies (Abs), clinical outcomes, and management in the long-term follow-up of eight out of fourteen patients with Graves' disease (GD) and encephalopathy associated with autoimmune thyroid disease (EAATD). The serum anti-thyroid Abs were assessed at the last follow-up vist, which took place at the reported follow-up month for each single patient (TG: thyroglobulin; TPO: thyroperoxidase; TSH: thyroid stimulating hormone; RAI: radioactive iodine).

## Discussion

Altered consciousness, involuntary movements (including tremor and myoclonus), seizures, cognitive impairment, focal neurological signs, ataxia, sensorial alterations, language impairment, symptoms of encephalitis (i.e., headache and nausea), and psychiatric alterations are the clinical manifestations of EAATD reported in GD patients. The hugely variable EAATD presentation in GD mirrors that occurring in HT patients. In both conditions, EAATD clinical manifestations can be fluctuating and relapsing. A rigorous statistical comparison of EAATD manifestations between the two groups is not realistic at the present stage due to the remarkable numerical difference of the two groups, the discrepancies in reporting the clinical data, and the virtually unlimited spectrum of the neurological and psychiatric symptoms and signs by which EAATD can manifest. All GD patients with EAATD and available hormonal data (12/14) had increased thyroid hormones at EAATD onset or few weeks before it, except one case of post-radioactive iodine hypothyroidism and one case of euthyroidism. The three patients which experienced a relapse of EAATD in the short-term presented with biochemical euthyroidism (two patients) or very mild iatrogenic hypothyroidism (one patient) when the encephalopathy reoccurred. Six cases of possible but not clearly defined GD, HT thyrotoxicosis, and non-GD-related hyperthyroidism concomitant to the occurrence of EAATD symptoms has been described as well [29-32]. On the other hand, a large number of HT patients presents with clinical or sub-clinical hypothyroidism at EAATD onset. Altogether, these data suggest that the hormonal changes are irrelevant in regard to the occurrence of EAATD that can develop independently from high, normal, or low thyroid hormone levels.

Anti-thyroid autoimmunity was active in all patients of the series described above, as documented by the anti-TPO Abs positivity. Anti-α-enolase and anti-neuronal Abs [9,10,33] are now getting relevance as possible diagnostic markers of EAATD but their use in the clinical practice is still limited. These auto-Abs have never been assayed in GD patients. Anti-TSH receptor Abs have been assayed by a radio-receptor method in the CSF of one GD patient only and were found negative. A link between enhanced anti-thyroid autoimmunity and the occurrence of EAATD seems to be a realistic hypothesis as suggested by the positive anti-TPO Abs. Indirectly, the hyperthyroidism caused by the anti-TSH receptor Abs at the time of the EAATD appearance in many GD patients confirms the same. However, the nature of the auto-Abs involved in the pathogenesis of EAATD and the mechanisms that cross anti-thyroid autoimmunity and encephalopathy are still undefined. The analysis of the CSF shows that an unspecific and mild inflammatory state can be detected in GD as well as HT patients with EAATD. The morphological analysis of the brain by the conventional radiological methods does not provide any specific findings but strongly contributes to the diagnosis because of the exclusion of other possible causes of the neurological or psychiatric symptoms. Interestingly, the hyperintense white matter changes occasionally detected at MRI appear to be reversible as previously reported also in case of HT [34]. In selected cases, imaging methods that study the vascular function of the brain, like magnetic resonance angiography and SPECT, can be useful tools for the diagnosis of EAATD. The findings from patients that underwent these procedures are compatible with the hypothesis of vasculitis or a vascular defect secondary to brain inflammation in the EAATD pathogenesis. The EEG patterns recorded in EAATD patients are unspecific, but a diffuse slowness of the background rhythm is the most common and characteristic report. Interestingly, a comparison of the laboratory, imaging, and electrophysiological findings of GD and HT patients with EAATD show no significant differences between the two groups (Table 3).

**Table 3:** Comparison between Hashimoto's thyroiditis and Graves' disease patients with encephalopathy associated with autoimmune thyroid disease.

	HT patients (n = 20)	GD patients (n = 14)	p
**+ serum anti-TPO Abs**	100%	100%	1

**+ serum anti-TG Abs**	60% (n = 10)	58% (n = 12)	1


**↑ CSF protein concentration**	85%	64% (n = 11)	0.2

**↑ CSF leukocytes count**	25%	45% (n = 11)	0.4

			

**normal MRI**	74% (n = 19)	62% (n = 13)	0.7

**abnormal EEG**	95%	92% (n = 13)	1

Statistical comparison of the most relevant immunological, cerebrospinal fluid (CSF), magnetic resonance imaging (MRI), and electroencephalogram (EEG) findings from a homogeneous series of Hashimoto's thyroiditis (HT) [12] and the series of Graves' disease (GD) patients with encephalopathy associated with autoimmune thyroid disease showing no differences between the two groups (TPO: thyroperoxidase; TG: thyroglobulin; Abs: antibodies).

Four cases of Graves' ophthalmopathy among the fourteen patients with GD and EAATD have been reported. One of them had an EAATD relapse during the long-term follow-up and developed also autoimmune hemophilia. He required an aggressive immunosuppressive treatment. This case is an exception in the series of GD patients with EAATD in regard to the gender (one of the two males), extra-thyroid GD manifestations, and therapeutic issues.

After the analysis of the patient series hereby reported, we are induced to postulate that the central nervous system should be enlisted among the possible extra-thyroid targets of GD and, thus, encephalopathy should be considered as a rare extra-thyroid manifestation of GD.

The majority of the patients had an impressive clinical and immunological improvement after the corticosteroid treatment, including the cases in which EAATD relapsed. This event was usually concomitant with the tapering or precocious withdrawal of the corticosteroids. We think that a one-year-long corticosteroid treatment represents a reasonable and advisable baseline therapeutic plan. Administration of a daily high-dose of corticosteroids during the acute phase of EAATD followed by a slow and careful dose tapering to withdrawal appears to be an effective therapeutic approach. In one case of the above series of GD patients, thyroidectomy after EAATD relapse led to a progressive and rapid improvement of the symptoms without any concomitant corticosteroid administration. This interesting report could suggest that thyroid antigens may play a direct role in the pathogenesis of EAATD by triggering the immunological reaction leading to encephalopathy.

## Conclusions

In conclusion, we show that GD can represent the background condition underlying EAATD. The clinical, immunological, radiological, electrophysiological, and therapeutic features of EAATD in GD do not differ from those of HT patients. The lack of differences in EAATD manifestations, findings, and outcomes between patients with GD and HT suggests that the diagnosis of EAATD should be considered in all patients with signs of encephalopathy of unknown origin and an autoimmune thyroid disease, independently by the functional status of the thyroid and the nature of the underlying autoimmune thyroid disease itself.

## Competing interests

The authors declare that they have no competing interests.

## Authors' contributions

GT conceived and coordinated the study, extracted and collected the data, interpreted the clinical data, and wrote the manuscript; YC, RS, MD, KK, GG, BIL, and CH acquired the clinical data, helped to interpret the same, and critically revised the manuscript; GM acquired the clinical data, helped to interpret the same, and helped to write the manuscript. All authors read and approved the final manuscript.

## Pre-publication history

The pre-publication history for this paper can be accessed here:

http://www.biomedcentral.com/1471-2377/10/27/prepub
